# Acne-induced pathological scars: pathophysiology and current treatments

**DOI:** 10.1093/burnst/tkad060

**Published:** 2024-04-05

**Authors:** Wanyu Xu, Dorsa Gholamali Sinaki, Yuchen Tang, Yunsheng Chen, Yixin Zhang, Zheng Zhang

**Affiliations:** Department of Plastic and Reconstructive Surgery, Shanghai Ninth People’s Hospital, School of Medicine, Shanghai Jiao Tong University, Shanghai 200011, China; Department of Plastic and Reconstructive Surgery, Shanghai Ninth People’s Hospital, School of Medicine, Shanghai Jiao Tong University, Shanghai 200011, China; Department of Plastic and Reconstructive Surgery, Shanghai Ninth People’s Hospital, School of Medicine, Shanghai Jiao Tong University, Shanghai 200011, China; Department of Burns and Plastic Surgery, Shanghai Institute of Burns Research, Ruijin Hospital Affiliated to Shanghai Jiao Tong University School of Medicine, Shanghai 200025, China; Department of Plastic and Reconstructive Surgery, Shanghai Ninth People’s Hospital, School of Medicine, Shanghai Jiao Tong University, Shanghai 200011, China; Department of Plastic and Reconstructive Surgery, Shanghai Ninth People’s Hospital, School of Medicine, Shanghai Jiao Tong University, Shanghai 200011, China

**Keywords:** Pathological scars, Acne, Inflammation, Fibrosis, Hypertrophic scars, Keloids

## Abstract

Acne is a common chronic inflammatory dermatosis that can lead to pathological scars (PSs, divided into hypertrophic scars and keloids). These kinds of abnormal scars seriously reduce the quality of life of patients. However, their mechanism is still unclear, resulting in difficult clinical prevention, unstable treatment effects and a high risk of recurrence. Available evidence supports inflammatory changes caused by infection as one of the keys to abnormal proliferation of skin fibroblasts. In acne-induced PSs, increasing knowledge of the immunopathology indicates that inflammatory cells directly secrete growth factors to activate fibroblasts and release pro-inflammatory factors to promote the formation of PSs. T helper cells contribute to PSs via the secretion of interleukin (IL)-4 and IL-13, the pro-inflammatory factors; while regulatory T cells have anti-inflammatory effects, secrete IL-10 and prostaglandin E2, and suppress fibrosis production. Several treatments are available, but there is a lack of combination regimens to target different aspects of acne-induced PSs. Overall, this review indicates that the joint involvement of inflammatory response and fibrosis plays a crucial role in acne-induced PSs, and also analyzes the interaction of current treatments for acne and PS.

Highlights

- In this article we reviewed the pathophysiological processes of pathological scars and their relationship with acne.
- The role of the joint involvement of inflammatory response and fibrosis in acne-induced pathological scars is discussed.
- The evidence for the use of common treatments and their effects in acne-induced pathological scars is reviewed.

## Background

Acne is a chronic inflammatory disease of the hair follicle and sebaceous gland associated with clinical manifestations of comedones, papules, nodules or cysts, and up to 95% of acne patients will develop scars. Depending on the tissue response, acne-induced scars can be classified as atrophic scars and pathological scars (PSs) [1, 2]. Classically, PSs are further defined by clinicians as a hypertrophic scar or keloid based on whether it grows beyond the original wound boundary. For PSs that are difficult to differentially diagnose, the Japan Scar Workshop scar scale can be used for classification and evaluation [3].

**Figure 1:**
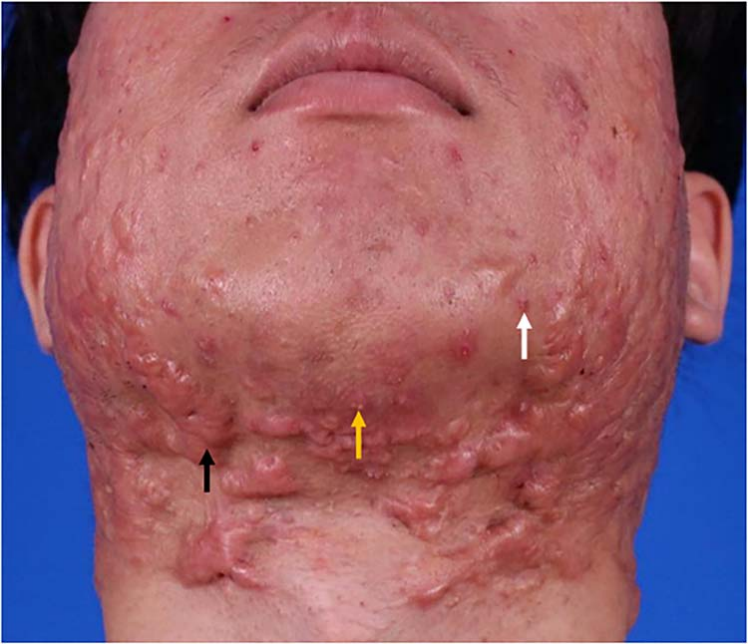
Clinical presentation of acne-induced pathological scars. Acne lesions, including papule (right arrow), pustule (middle arrow) and pathological scar (left arrow) on the face

Clinically, the lesions of acne patients often exhibit polymorphic characteristics [4], such as the simultaneous presence of papules and PSs (Figure 1). Acne is the main cause of PSs [5], especially in patients with multiple PSs on the chest, back and jaw. However, the pathophysiology of acne-induced PS formation is still unclear, and therefore there is a lack of effective prevention and treatment methods for these scars, resulting in the recurrence of this condition in patients [6]. Therefore, clarifying the relationship between acne and PSs is expected to identify more effective clinical treatment strategies for acne-induced PSs. In this focused literature review, the mechanisms of pathogenic and therapeutic modulation of acne-induced PS are examined, and recommendations for future research directions and clinical treatments are provided.

## Review

### Epidemiology

Among patients with acne scars, ~23.80–26.94% develop PSs [7, 8] and the incidence increases with the severity of acne [9]. However, compared to atrophic scars, PSs account for a smaller proportion of cases [4], and therefore their diagnosis and treatment are often neglected.

The average age of patients with acne-induced PSs is ~21 years. Although acne tends to be more severe in men than in women [10], there is no significant gender bias in the occurrence of PS. Acne frequently occurs on the capillary-rich face, chest and back, and has prevalence rates of 92, 45 and 61%, respectively; scarring occurs during the healing of active acne [4]. A retrospective study showed that PSs tend to occur in areas of greater tension, such as the anterior chest, scapula and jawline. Tension in the skin may lead to long-term, recurrent inflammation of the dermal reticular layer and the formation of abnormal amounts of blood vessels, collagen and nerve fibers, which subsequently cause the development of PSs and the associated clinical symptoms [7, 11].

There is genetic susceptibility to the development of PSs, but there is also genetic heterogeneity and differences in the degree of expression; pathogenesis involves multigene regulation, which is extremely complex, so most of these conditions do not show familial trends but tend to have a scattered onset. However, some genes, such as the neuronally expressed developmentally downregulated 4 (NEDD4) gene, are thought to influence the severity of PSs by enhancing the proliferation and invasiveness of fibroblasts and activating the transforming growth factor-β (TGF-β)/catenin signaling pathway [12, 13]. The wingless-related integration site family member 10A (WNT10A) gene has been implicated in genetic susceptibility to severe acne vulgaris and in aspects of wound healing and fibrosis by regulating collagen synthesis and contributing to keloid proliferation by regulating telomerase [14]. The CC genotype of matrix metalloproteinase (MMP)-2 (−1306 C/T) is a specific immunological factor that has also been shown to be significantly associated with acne-induced PSs [15].

### Pathophysiology

**Figure 2:**
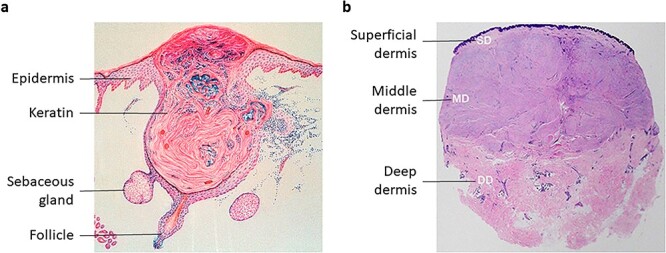
Characteristics of lesions of acne and acne-induced pathological scars. Histological images of an inflammatory acne lesion (**a**) and a pathological scar lesion (**b**) are shown. The sebum production of sebaceous gland, the buildup of keratin and the accumulation of inflammatory cells are the typical features of acne. However, when it becomes a pathological scar, large numbers of fibroblasts and collagen bundles occupy the area of lesion. Reproduced with permission from [16] (Copyright 2023 Shanghai China) and [17]

The exact pathophysiology of acne-induced PSs is unknown. According to clinical evidence, a local lesion always develops in the pilosebaceous unit as a small papule associated with inflammation, but it can finally become a PS (Figure 2). Therefore, current studies suggest the joint involvement of the inflammatory response and fibrosis.

**Figure 3:**
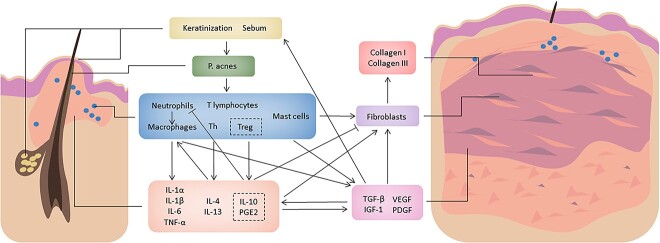
Pathophysiological processes in acne-induced pathological scars formation. The development of acne-induced pathological scars depends on the joint involvement of abnormal keratinization, overproduction of sebum, proliferation of bacteria, inflammatory response and fibrosis. *P. acnes Propionibacterium acnes*, *Th* helper T cells, *Treg* regulatory T cells, *IL-1α* interleukin-1α, *TNF-α* tumor necrosis factor-α, *PGE2* prostaglandin E2, *TGF-β* transforming growth factor-β, *IGF-1* insulin-like growth factor-1, *VEGF* vascular endothelial growth factor, *PDGF* platelet-derived growth factor

The chronic inflammatory response stimulates the activation of fibrosis-related cytokines and signaling pathways. Moreover, fibrosis inhibits the dissipation of inflammation, and these processes complement each other to form a vicious cycle, which ultimately leads to the development of acne-induced PSs and prolonged clinical features (Figure 3) [18].

### Inflammation cascades

Acne occurs as a result of impaired sebum drainage, and as the disease progresses, *Propionibacterium acnes* (*P. acnes*) infection and other factors induce an inflammatory response leading to acne. PSs are thought to be associated with an inflammatory response and are frequently observed in acne conglobata, hidradenitis suppurativa (acne inversa) and perifolliculitis capitis abscedens et suffodiens [19]. Some PSs also discharge pus due to internally infected cysts, and internal cysts arise due to the engulfment of hair follicles. Several investigators have suggested that inflammation stimulates collagen synthesis and that the degree of inflammation is positively correlated with scar size [7]. Therefore, acne with a lesser degree of inflammation affects the dermis at a shallower depth and produces smaller scars. Other studies have shown that PSs do not form in the early stages of acne and that damage such as skin inflammation can only lead to PS formation if it reaches the reticular layer, which also exhibits accelerated angiogenesis and collagen accumulation [11]. Fibrosis is deeper in acne-induced keloids than in hypertrophic scars [7]. The combined involvement of microorganisms and inflammatory cells and factors leads to the development of inflammation and sets the stage for an abnormal fibrotic process.

#### P. acnes

*P. acnes* is a microorganism that is stably distributed in oily skin areas such as the face and anterior chest [20, 21]. It is closely associated with the development of acne [22]. In the early stages of acne, epithelial keratinization of follicular sebaceous glands and hypersecretion by sebaceous glands create a suitable environment for the proliferation of anaerobic microorganisms such as *P. acnes* (1). After proliferating in hair follicles, *P. acnes* induces inflammatory responses by acting on naive CD4+ T lymphocytes, converting them into T helper (Th)17 cells, which release interleukin (IL)-17 (10), or stimulating Langerhans cells, keratinocytes and sebocytes via Toll-like receptor 2 (TLR-2), which leads to increases in IL-12, IL-8, IL-6, interferon-c and tumor necrosis factor-α (TNF-α) [23, 24]. Furthermore, some resistant strains may chronically stimulate the innate immune system, resulting in extensive inflammation [25] and the destruction of follicular walls through the production of lipase, hyaluronidase and protease, ultimately contributing to PS formation [19]. A study by Canesso MC *et al*. found significantly faster wound healing and no scarring in mice after the removal of commensal microflora, demonstrating that microorganisms may play an important role in scar development [26].

#### Inflammatory cells

Histological studies of PSs have revealed the presence of inflammatory cells, including neutrophils, macrophages, T lymphocytes and mast cells. Early in acne inflammation, neutrophil recruitment activates macrophages, which regulate the migration and adhesion of inflammatory cells by releasing inflammatory factors such as IL-1α, IL-1β, IL-6 and TNF-α [27], and cytokines such as insulin-like growth factor-1 (IGF), TGF-β and platelet-derived growth factor (PDGF), which promote the differentiation of fibroblasts to myofibroblasts and the synthesis and secretion of extracellular matrix, which in turn leads to PS formation [28].

The activation and migration of T lymphocytes occur later than that of macrophages, and Th cells and regulatory T (Treg) cells play an important role in scar formation. Th cells mediate the local inflammatory response in PSs and secrete proinflammatory cytokines such as IL-4 and IL-13, which also exhibit strong pro-fibrotic effects. Studies have shown that the proliferation of Th17 cells is regulated by TGF-β, and the IL17/Th17 pathway is associated with the inflammatory reaction in acne lesions [29]. Some factors, such as vitamin A and vitamin D, can modulate Th17-mediated acne [30]. Tregs, which are functionally antagonistic to Th cells, have anti-inflammatory effects, secrete IL-10 and PGE2, directly inhibit neutrophil chemotaxis and infiltration, reduce the release of inflammatory factors and suppress fibrosis [31]. When Th cells and Treg cells are imbalanced, they can cause an excessive local immune-inflammatory response. TGF-β can heavily affect the differentiation of T lymphocytes, favoring Th17 instead of Treg differentiation through direct or indirect effects [32].

Mast cells directly activate fibroblasts via gap junctions [33]; on the other hand, these cells release histamine, heparin and various chemokines through degranulation to participate in scar formation. In scar tissue, mast cells secrete increased levels of chemokines that promote fibroblast proliferation and collagen synthesis via TGF-β1/Smad activation. The release of histamine and heparin and other factors leads to the proliferation of microvascular endothelial cells and the symptoms of pruritus, creating the congested appearance and discomfort associated with PS formation [12]. It has been shown that histamine significantly upregulates collagen synthesis in fibroblasts, that serum levels of histamine are significantly higher in patients with PS than in the normal population and that the amount of histamine released by mast cells originating from PS tissue is significantly higher than that released by normal mast cells [34].

#### Inflammatory factors

As the inflammatory phase of acne is prolonged, immune cells continue to release a variety of cytokines associated with the inflammatory response. In acne-induced PS formation, the expression of anti-inflammatory cytokines such as IL-10 decreases and the expression of proinflammatory cytokines such as TNF-α increases; these factors have been shown to play crucial roles in the development of acne-induced PSs [19]. Studies have also shown that TNF-α may be directly involved in PS formation, while IL-4 and IL-13 result in the production of a series of growth factors, including TGF-β, IGF, vascular endothelial growth factor (VEGF) and PDGF, through the activation of macrophages, all of which are involved in scar formation to varying degrees. IL-6 can increase extracellular matrix secretion. IL-6 also affects the secretion of MMP-1 and MMP-3 precursor proteins to promote PS formation.

IL-10, which has anti-inflammatory effects can directly inhibit collagen synthesis and secretion and inhibit autophagy in PS fibroblasts through IL-10–IL-10 receptor signaling and transcriptional activators and the IL-10–Akt-mammalian rapamycin target protein pathway. Scar fibroblasts overexpressing IL-10 can effectively inhibit inflammatory responses and scar formation [35]. One study showed that IL-10-knockout mice had an increased inflammatory response to trauma and significant keloid proliferation compared to wild-type mice [36].

### Abnormal activated fibroblasts and dysregulation of fibrosis

The proliferation of fibroblasts and deposition of extracellular matrix are key to PS formation. Fibroblasts are the key effector cells involved in PS formation and secrete the main components of the extracellular matrix: type I and type III collagen. During the proliferative phase of injury repair, fibroblasts gradually proliferate and differentiate into functional secretory myofibroblasts [37]. The persistent inflammatory response to acne activates relevant signaling pathways, which stimulate fibroblast proliferation and extracellular matrix secretion, ultimately leading to PS formation. In current studies on PSs, cytokines and signaling pathways associated with fibrosis include IGF-1/IGF-1 receptor (IGF-1R), TGF-β/Smad, VEGF, PDGF and others. Among them, IGF-1, IGF-1R and downstream mechanistic target of rapamycin (mTOR) play key roles in the development of acne and the formation of acne-induced PSs.

#### IGF-1

IGF-1 is a key player in acne pathogenesis, and the IGF-1-induced PI3K/Akt/FoxO1/mTOR C1 pathway is considered to be the most important pathway leading to acne pathogenesis [38]. Moreover, IGF-1 can promote the proliferation and differentiation of sebocytes and IL-1β production [19], increase sebum through sterol regulatory element-binding protein (SREBP) [1] and interact with androgens to promote the development of acne [39]. Studies have shown that elevated serum levels of IGF-1 are positively correlated with the number of acne lesions in women [10], and lowering IGF-1 levels may prevent the development of acne [39]. In studies on the mechanism of dietary factors that affect acne, it was also shown that hyperinsulinemia caused by high glycemic index (GI) diets triggers acne by increasing the ratio of IGF-1 to insulin-like growth factor binding protein-3 through the insulin/IGF-1 signaling pathway, thereby enhancing the effect of IGF-1 [10]; conversely, low GI diets significantly reduced acne severity and improved insulin sensitivity [25]. IGF-1 modulators have been reported to be effective treatments for acne [38]. The IGF-1/IGF-1R pathway is associated with many fibrotic diseases. IGF-1R was highly expressed in acne-induced PS tissues, and it supports scarring by increasing fibroblast invasiveness and inhibiting their apoptosis; therefore, previous studies suggested that targeting IGF-1R in fibroblasts may help prevent scar formation [40]. However, for acne-induced PS formation, focusing on the target of IGF-1 may achieve better results.

#### TGF-β

TGF-β is a cytokine that is closely related to fibrosis and inflammation and stimulates the proliferation and activation of dermal fibroblasts and their conversion into myofibroblasts, which produce large amounts of extracellular matrix components [41] and act on inflammatory cells to regulate cell proliferation, differentiation and migration [42]. Severe acne can lead to scarring, and overexpression of TGF-β can further lead to PS formation [43]. TGF-β includes five isoforms, among which TGF-β1 has the highest proportion and strongest activity, upregulates α-smooth muscle actin (α-SMA) and collagen expression, and promotes extracellular matrix synthesis and angiogenesis. The expression of TGF-β1 and TGF-β3 was significantly elevated in acne-induced PS tissue.

Many previous studies have shown that TGF-β has an important role in PS formation [44]. In acne lesions, TGF-β is highly upregulated and affects sebocytes and keratinocytes, leading to excessive lipogenesis and abnormal proliferation of keratinocytes [29, 45]. Caveolin-1 (Cav-1), which is a strong negative regulator of TGF-β expression, is an important factor affecting the pathophysiological process of acne, and low Cav-1 expression is often associated with excessive cell proliferation and inflammation, is closely related to scar formation and is expected to be a target for acne treatment [46], providing a possible direction for the study of acne-induced PS formation. In addition, there is a correlation between the TGF-β/Smad signaling pathway, which is closely related to scar fibrosis, and acne [42]. In PSs, activated TGF-β binds to TGF-β type II (TβR-II) and activates TβR-I, which induces small mothers against decapentaplegic (Smad)2/3 phosphorylation, forming a Smad2/3-Smad4 binding transcriptional complex that translocates into the nucleus and acts on the corresponding target genes [29]. Fibrosis can be attenuated by inhibiting TGF-β and regulating the expression of Smads. A study of a mouse model of auricular acne showed that taraxerol, which has anti-inflammatory effects, can regulate the expression of inflammatory factors and alleviate the inflammatory response through the TGF-β/Smad pathway to treat acne. Some studies have shown that a specific inhibitor of Smad3 can improve fibrosis and inflammation by inhibiting the TGF-β/Smad3 signaling pathway [47], but this has not been observed in studies of acne-induced PSs. The mitogen-activated protein kinase (MAPK) pathway has been reported to be involved in the transcription of TGF-β, and p38 is involved in the phosphorylation of Smad2/3 in keloid fibroblasts, whereas extracellular signal-regulated kinase (ERK) and c-Jun N-terminal kinase (JNK) promote translocation of Smad2/3/4 complex, which in turn regulates the expression of related genes, such as plasminogen activator inhibitor-1 (PAI-1) [48]. Stimulation with *P. acnes* enhances the phosphorylation of JNK and p38, but fibroblast growth factor 21, which is an anti-inflammatory factor, inhibits *P. acnes*-induced activation of MAPK signaling in HaCaT cells [49]. Another study showed that the receptor tyrosine kinase inhibitor, nintedanib, inhibited keloid fibroblast function by inhibiting the MAPK pathway components p38, ERK and JNK, as well as the phosphorylation of Smad2 and Smad3, in a dose-dependent manner [50].

#### Other related factors

In addition to inflammatory and fibrosis-related factors, the abnormal vascular manifestations of acne-induced PSs have recently begun to receive attention. Studies suggest that VEGF and PDGF [11], which are closely related to blood-vessel formation and function, may be involved in the progression of acne to PS. VEGF influences the inflammatory process in acne by increasing the permeability of the vessel wall and promoting monocyte aggregation. VEGF levels are lower and there is less angiogenesis in scarless wounds than in fibrotic wounds. The addition of exogenous VEGF can convert the scarless phenotype into a PS phenotype and hinder the inhibitory effect of glucocorticoid injections on fibroblast proliferation in PSs. Neutralizing VEGF in wounds can improve scarring [51, 52]. These findings suggest that VEGF supports scarring. PDGF is also associated with PSs. Chen *J et al*. found elevated PDGF expression in PSs, while captopril significantly reduced PDGF mRNA and protein expression in fibroblasts [53].

### Treatment

**Figure 4:**
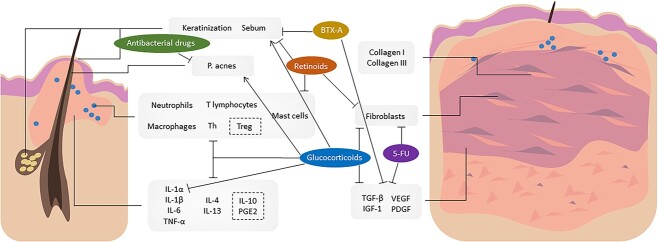
Effect of different medications in acne-induced pathological scars treatment. Common medications, such as retinoids, glucocorticoids, BTX-A, antibacterial drugs and 5-FU, have tangled interactions in acne and pathological scars treatment. *BTX-A* botulinum toxin type A, *5-FU* 5-fluorouracil, *P. acnes Propionibacterium acnes, Th* helper T cells, *Treg* regulatory T cells, *IL-1α* interleukin-1α, *TNF-α* tumor necrosis factor-α, *PGE2* prostaglandin E2, *TGF-β* transforming growth factor-β, *IGF-1* insulin-like growth factor-1, *VEGF* vascular endothelial growth factor, *PDGF* platelet-derived growth factor

Current treatments for acne and PS include medication, light therapy and laser therapy (Table 1). Among them, the application of various types of drugs plays an important role (Figure 4).

**Table 1:** Summary of common treatments and their effects on acne and pathological scars

**Treatments**		**Represented medicines**	**Effects**	**Pathological scars**
			**Acne**	
Medication	Retinoids	Adapalene, Isotretinoin	Anti-inflammatory, inhibit sebaceous gland secretion, regulate abnormal keratinization and improve the environment of hair follicles	Inhibit fibroblast proliferation and reduce collagen fiber production
	Glucocorticoids	Tretinoin	Inhibit the conversion and expression of TGF-β1, reduce the inflammatory response	Inhibit fibroblast proliferation and induce apoptosis, and reduce collagen synthesis; stimulate sebaceous gland cells, increase *P. acnes*, and free fatty acids
	Botulinum toxins	BTX-A	Inhibit glandular secretion	Reduce local tension, downregulate TGF-β1 expression, inhibit fibroblast proliferation, and regulate collagen deposition
	Antibacterial drugs	BPO, Minocycline	Inhibit or kill *P. acnes*	NR
	Cytotoxic drugs	5-FU	Improve the onset of acne	Inhibit thymidylate synthase, affect fibroblast proliferation and block TGF-β-mediated type I collagen gene expression, reducing collagen production
Light therapy and laser therapy	Intense pulsed light		Destroys the sebaceous glands and inhibit the proliferation of *P. acnes*	Targets oxyhaemoglobin and pigment chromophores, treating the hyperpigmentation and erythema problems
	Photodynamic therapy		Produces reactive oxygen species to damage sebaceous glands and reduce *P. acnes*, reduces inflammatory markers	Generates reactive oxygen species to cause necrosis and apoptosis of fibroblasts, down-regulates collagen I, α-SMA and TGF-β1 and reduces the LC3 II/I ratio
	Pulsed dye laser		Targets hemoglobin to accelerate the fading of red papules, releases anti-inflammatory cytokines and eliminates bacteria	Acts on hemoglobin, improving scar erythema
	Fractional CO_2_ laser		Causes photo-thermolysis of sebaceous glands	Breaks down abnormal scars, releases tension and upregulates MMPs

*TGF-β* transforming growth factor-β, *P. acnes Propionibacterium acnes*, *BTX-A* botulinum toxin type A, *BPO* benzoyl peroxide, *NR* none reported, *5-FU* 5-fluorouracil, *α-SMA* α-smooth muscle actin

#### Retinoids

Retinoids are central to acne treatment and mainly include topical and oral treatments. Topical retinoids, such as adapalene, have catabolic, anti-pimple and anti-inflammatory effects, reduce hyperpigmentation, have a collagen-promoting effect that helps rebuild the papillary matrix [25] and are commonly used to treat mild to moderate acne. In contrast, for moderate to severe acne and mild acne (especially jaw acne) that is already associated with scarring, has a long duration or does not respond to topical treatment, systemic treatment with drugs, such as oral retinoids, is typically required [25, 54]. Isotretinoin, which is the first choice among oral retinoids, significantly inhibits sebaceous gland secretion, regulates abnormal keratinization of follicular sebaceous ducts and improves the anaerobic environment of hair follicles. Furthermore, studies have shown that oral isotretinoin can inhibit fibroblast proliferation and reduce collagen fiber production in the early stage of scar formation, which has a certain effect on preventing scar formation. However, these agents are not widely used in the clinical treatment of acne-induced PS, even though current clinical observations have shown that it is safe to perform invasive therapeutic procedures, including laser therapy and surgery while taking oral isotretinoin or just after stopping treatment [55, 56]. Thus, the application of retinoids should be included in the comprehensive clinical treatment of acne-induced PSs.

#### Glucocorticoids

Glucocorticoids are widely used to treat PSs and in a proportion of patients with severe acne to inhibit the conversion and expression of TGF-β1, reduce the inflammatory response, inhibit fibroblast proliferation, induce apoptosis and reduce collagen synthesis. Currently, these agents are mainly used to treat acne and PSs by oral or local injections, respectively.

Oral low-dose glucocorticoids (e.g. prednisone, dexamethasone) are recommended for acute flare-ups and short-term treatment of severe acne [57]; injections of glucocorticoids (e.g. tretinoin) are currently one of the most commonly used treatments for PS, are often used in combination with cytotoxic drugs and have significant clinical effects [58].

However, glucocorticoids have side effects such as skin atrophy, capillary dilation, infection and hypopigmentation. It has also been shown that glucocorticoids can stimulate the proliferation of sebaceous gland cells, increase *P. acnes* and increase free fatty acid concentrations, causing the development of acne [59]. Treatment of keratin-forming cells with glucocorticoids can increase the expression of TLR-2 receptors and increase the release of TNF-α and IL-1α, thus playing an important role in the formation of acne [60]. Therefore, in acne-induced PSs, there may be situations where the use of long-term, higher doses of glucocorticoids may lead to new acne or exacerbate primary acne.

#### Botulinum toxins

Botulinum toxin type A (BTX-A) acts on peripheral motor nerve endings and inhibits acetylcholine release, relaxes and paralyzes muscle fibers, reduces local tension, downregulates TGF-β1 expression, inhibits fibroblast proliferation and regulates collagen deposition, thereby preventing and treating PSs [61, 62]. A meta-analysis showed that BTX-A was effective in preventing PS formation in the maxillofacial and cervical regions [63]. Moreover, BTX-A can inhibit glandular secretions by blocking the release of parasympathetic neurotransmitters. Hong Suzhuang *et al*. reported that the acute inflammatory response dissipated after the injection of BTX-A around the lesion in patients with inflammatory acne-induced PSs [64]. However, the recurrence of deep inflammatory reactions was seen in all cases to varying degrees, and multiple injections were required in all cases [64]. Compared to glucocorticoids, BTX-A has fewer associated serious adverse events [65, 66] and a higher safety profile [67]. A previous clinical study showed that treatment of acne-induced keloids with 10 U/100 cm^2^ BTX-A in combination with conventional steroid injections reduced sebum secretion and keloid recurrence. Moreover, there were no side effects other than pain and bleeding at the injection site [68].

#### Other related medications

In addition to these drugs, antibacterial drugs for the treatment of acne and cytotoxic drugs for treating PS are also widely used in clinical practice. The main antibacterial drugs used in the clinical treatment of acne include topical benzoyl peroxide (BPO) gel, clindamycin hydrochloride gel and fusidic acid cream, as well as oral minocycline and doxycycline. These drugs mainly inhibit or kill *P. acnes* and clearly inhibit acne. However, in acne patients who have developed PSs, there is no direct evidence that the use of antibacterial drugs can benefit scarring.

The main cytotoxic drug currently used to treat of PSs is 5-fluorouracil (5-FU), which inhibits thymidylate synthase, affects fibroblast proliferation and blocks TGF-β-mediated type I collagen gene expression, thereby reducing collagen production. Some studies have shown that subcutaneous injection of low concentrations of 5-FU can rapidly improve the onset of acne in and around PSs. It is also effective in treating simple acne vulgaris [69].

#### Light and laser therapy

While medication plays an important role, light and laser therapies such as intense pulsed light (IPL), photodynamic therapy (PDT), pulsed dye laser (PDL) and fractional CO_2_ laser have been increasingly emphasized as important elements in the treatment regimen for acne-induced PS.

IPL treats acne through selective photothermolysis, targeting the blood vessels of the sebaceous glands to destroy them and triggering photoexcitation of porphyrins to produce reactive oxygen species that inhibit the proliferation of *P. acnes* with minimal discomfort and rapid recovery time [70, 71]. Several previous studies of patients with moderate to severe acne showed that multiple sessions of IPL alone at 400–700 nm significantly improved the severity of acne by ~81–88% [72–74]. On the other hand, IPL is mainly used in combination with other treatment modalities to treat PSs. IPL effectively targets oxyhemoglobin and pigment chromophores, thereby treating the hyperpigmentation and erythema problems associated with PSs [75]. A network meta-analysis that included 25 trials with a total of 1688 participants showed that the combination of IPL and CO_2_ laser was the most effective (96.43%) intervention for PSs [76].

PDT, which involves activation by photosensitizers such as 5-aminolevulinic acid (5-ALA), produces reactive oxygen species and free radicals to damage sebaceous glands and reduce *P. acnes*, and reduce TLR-2 and TLR-4 in sebaceous glands and the epidermis [77]. One study reported that the use of 20% ALA-PDT was even better than the drug combination used for patients with moderate inflammatory facial acne [78]. 5-ALA-PDT has been used to treat patients with PSs by generating enough reactive oxygen species to cause efficient necrosis and apoptosis in fibroblasts while downregulating the gene expression levels of collagen I, α-SMA and TGF-β1 and reducing the light chain 3 (LC3) II/I ratio, thus effectively improving the appearance and thickness of PSs. Furthermore, a number of studies are underway to further improve the efficacy of PDT by modifying photosensitizers, such as through the construction of 5-ALA–hyaluronic acid complexes, and the combination of hyaluronidase and metformin [79, 80].

The reduction of acne by PDL is achieved by targeting hemoglobin to accelerate the fading of red papules and stimulating T cells to release anti-inflammatory cytokines and eliminate bacteria [81]. Previous studies have shown a 57–83% improvement in PSs after several PDL treatments [82]. The therapeutic effect of PDL on PSs is mainly on hemoglobin within microvessels, thereby improving the vascularity and blood perfusion of the scars, thus making PDL effective in improving scar erythema [83–86]. However, PDL may still improve scar thickness by downregulating TGF-β1 or other growth factors, although it provides fewer improvements in scar thickness than CO_2_ fractional laser [87–90].

The fractional CO_2_ laser is the most commonly used ablative laser in the treatment of acne-induced PSs. It causes photothermolysis of the sebaceous glands by ablating the epidermis and some of the dermal tissue and coagulating the surrounding tissue [91]. After one to two CO_2_ fractional laser treatments, the number of active acne lesions can be reduced by 26–50% [92]. Furthermore, the microperforations caused by fractional CO_2_ lasers allow for abnormal scar breakdown, scar tension release and the upregulation of MMPs, thereby reducing scarring [93–95]. Conditions were compared before and after fractional CO_2_ laser treatment, and PSs improved in terms of flexibility, height, pain and itching [96, 97].

The best light or laser therapy for the clinical treatment of acne-induced PSs remains unclear. Although the fractional CO_2_ laser can significantly improve scarring, it causes more pain and requires a longer recovery time [98]; IPL is quite comfortable but has insufficient evidence for treating PS alone; PDL is more effective at improving erythema; and PDT has the best evidence for treating acne and may be more effective at controlling the overall skin condition of the lesion, thereby reducing the rate of new scarring. There is a growing body of research on combinations of light or laser modalities, but there is a need for larger studies to identify the most appropriate light or laser treatment options for these patients.

#### Others

For acne-induced hypertrophic scars and keloids, surgery, radiation therapy, compression and adipose-derived stem cell therapy can also be used. Treatments for extensive inflammatory keloids are limited. Surgery and postoperative radiation therapy are two possible options [19]. A study that included 141 patients with acne-induced keloids showed that after surgery combined with localized radiation therapy and management, 89.4% of the patients were free of recurrence [99]. The key to surgery is to minimize tension, which can be accomplished through the intraoperative use of tension-reducing sutures, z-plasty and local flap transfers. For keloids, surgery should always be used in conjunction with radiation therapy, since surgical excision alone can have a recurrence rate of 45–100% [100].

Radiation therapy, including superficial X-rays and electron beams, has been widely used to treat or prevent keloids by inhibiting angiogenesis, suppressing inflammation and inhibiting fibroblast proliferation [101]. The results of a recent meta-analysis showed that the recurrence rate of surgery combined with radiation therapy was much lower than that of radiation therapy alone, suggesting that most patients should be treated with the combination, whereas radiation monotherapy is reserved only for elderly patients or for patients with large keloids that cannot be excised and sutured [102]. The maximum biologically effective dose (BED) for keloids is 30 Gy and site-specific postoperative radiation therapy regimens are as follows: (1) for keloids on the anterior chest wall, scapular region and suprapubic region, 20 Gy should be administered in four divided doses over 4 days (BED = 30 Gy); (2) for earlobe keloids, 10 Gy should be administered in two divided doses over 2 days (BED = 15 Gy); and (3) for keloids in other areas, 15 Gy should be administered in three divided doses over 3 days (BED = 22.5 Gy) [103].

In addition, a potential option for the prevention and treatment of acne-induced PSs is adipose-derived stem cell therapy. Studies have shown that adipose-derived stem cells can modulate the inflammatory response by secreting different bioactive substances and can inhibit α-SMA expression, thereby inhibiting fibroblast proliferation and reducing fibrosis [104, 105].

## Conclusions

The pathophysiological processes of acne-induced PS formation are complicated. Following abnormal epithelial keratinization and impaired sebum drainage, the proliferation of *P. acnes* induces an inflammatory response. Neutrophil-activated macrophages overproduce IL-1α, IL-1β, IL-6 and TNF-α, and secrete TGF-β, PDGF and IGF through the IGF-1/IGF-1R and TGF-β/Smad pathways, leading to fibrosis, abnormal vascular manifestations and further increases in inflammatory factors. Moreover, Th cells contribute to PS formation via the secretion of IL-4 and IL-13. Based on the results of research, IGF-1 related to insulin and a specific inhibitor of Smad3, and IL-17 antibodies related to inflammatory pathways, are expected to become targets for the prevention and treatment of acne-induced PSs, providing potential directions for new drug development. Acne-induced PSs require a comprehensive treatment strategy. However, current use of medications for PSs is still mainly limited to local injections and may not be sufficient to control the overall condition of patients. The application of retinoids and glucocorticoids for systemic treatment, the reformulation of existing drugs and combinations with light or laser therapy are directions for future clinical research. Future studies will continue to search for a safe permanent cure for acne-induced PS.
